# Complete mitogenomes characterization and phylogenetic analyses of *Ceratophyllus anisus* and *Leptopsylla segnis*

**DOI:** 10.3389/fvets.2023.1218488

**Published:** 2023-06-16

**Authors:** Yafang Liu, Bin Chen, Xinyan Lu, Dandan Jiang, Tao Wang, Ling Geng, Quanfu Zhang, Xing Yang

**Affiliations:** ^1^Integrated Laboratory of Pathogenic Biology, College of Preclinical Medicine, Dali University, Dali, China; ^2^School of Public Health, Dali University, Dali, China; ^3^Department of Gastroenterology, Clinical Medical College and The First Affiliated Hospital of Chengdu Medical College, Chengdu, Sichuan, China

**Keywords:** fleas, *Ceratophyllus anisus*, *Leptopsylla segnis*, mitogenome, phylogenetic

## Abstract

Fleas are one of the most common ectoparasites in warm-blooded mammals and an important vector of zoonotic diseases with serious medical implications. We sequenced the complete mitochondrial genomes of *Ceratophyllus anisus* and *Leptopsylla segnis* for the first time using high-throughput sequencing and constructed phylogenetic relationships. We obtained double-stranded circular molecules of lengths 15,875 and 15,785 bp, respectively, consisting of 13 protein-coding genes, 22 transfer RNAs, 2 ribosomal RNAs, and two control regions. AT-skew was negative in both *C. anisus* (−0.022) and *L. segnis* (−0.231), while GC-skew was positive in both (0.024/0.248), which produced significant differences in codon usage and amino acid composition. Thirteen PCGs encoding 3,617 and 3,711 codons, respectively, isoleucine and phenylalanine were used most frequently. The tRNA genes all form a typical secondary structure. Construction of phylogenetic trees based on Bayesian inference (BI) and maximum likelihood (ML) methods for PCGs. The results of this study provide new information for the mitochondrial genome database of fleas and support further taxonomic studies and population genetics of fleas.

## Introduction

Fleas are the most notorious blood-sucking ectoparasites that transmit a variety of zoonotic pathogens to infect domestic and wild animals (1). Fleas can host a wide range of species, including human, domestic pets, rodents, and ungulates, resulting in a shortened host lifespan, while ecologically related hosts might share flea species, which increases the public health impact of fleas and causes serious economic losses (2). Fleas play a significant role in maintaining the plague’s natural foci (3). Several important pathogens have been identified in fleas, including plague, epidemic hemorrhagic fever, tularemia, leptospirosis, and murine typhus (4). Due to the significant medical, veterinary, and economic value of fleas, increasing attention has been placed on diseases transmitted by fleas, which currently cost global more than $15 billion annually (5). However, to date, phylogenetic relationships and clear classification among fleas have remained ambiguous, which has greatly hindered the control of fleas.

*Ceratophyllus anisus* belonging to the Siphonaptera order, Ceratophyllidae family, *Ceratophyllus* genus, is the main vector of plague. *C. anisus* can parasitize *Rattus norvegecus*, *Rattus tanezumi*, *Apodemus agrarius*, and *Crocidura attenuata*, and occasionally human. *C. anisus* has been found in the former Soviet Union, Korea, and Japan, and is most widely distributed in Yunnan Province, China (6). *Leptopsylla segnis*, in the genus *Leptopsylla* of the family Leptopsyllidae, has been reported in Libya and Cyprus and is common in numerous provinces of China, where wild rodents are most often infested by it (7, 8). Due to the large number of pathogens carried by fleas and the low resolution of traditional taxonomic features for fleas, the choice of a rapid and accurate identification method is necessary for the control of fleas and flea-borne diseases (9).

With the development of molecular and phylogenetic studies, the mitochondrial genome has been used in ectoparasites taxonomy, systematics, and population genetics, which to some extent make up for the limitations of traditional morphology (10, 11). The mitochondrion, the organelles of eukaryotic cells that maintain the structure of life, possess their genetic material and can replicate autonomously outside the nucleus. Mitochondrial DNA (mtDNA) has the advantages of simple structure, little recombination, fast evolution rate, and maintains inheritance in the process of evolution, which makes it an effective tool for studying species identification, relatedness, and phylogeny (12, 13). However, the mitochondrial genome data of fleas is still extremely scarce, and the phylogenetic relationship has not been established, which is a major obstacle to the prevention and control of fleas and flea-borne diseases.

This study is the first to sequence and analyze the mitochondrial genomes of *C. anisus* and *L. segnis*, with the aim of contributing to their correct identification and classification, enriching the mitochondrial genome database of fleas, facilitating the prevention and control of diseases caused by them to minimize the risk to hosts and humans, and providing new and useful markers for further species identification and molecular epidemiological studies. Genetic markers for further species identification and molecular epidemiological studies. We also studied the phylogenetic relationships with the gene sequences of other flea species in NCBI, which provides an important basis for population genetic, phylogenetic and evolutionary analysis.

## Materials and methods

### Sample collection and DNA isolation

The *C. anisus* samples (two females and one male) used in this study were collected in June 2022 from wild *R. tanezumi* in Laojun Mountain, Lijiang City, Yunnan Province of China (26°53′N, 99°58′E). The adult fleas *L. segnis* specimens were collected in August 2022 in Jianchuan, Yunnan Province from *R. norvegicus* (26°57′N, 99°90′E) (one female and one male). Preliminary identification of the collected flea specimens based on morphological diagnostic features (14). One specimen each was selected for subsequent DNA extraction and mitochondrial genome sequencing, while the others were placed in the Museum of Parasitology, Dali University, under voucher numbers DLUP2206 and DLUP2208, respectively. Specimens for experiments were rinsed in 0.9% saline, fixed in 96% alcohol and stored at −80°C until used for DNA extraction (11). DNA extraction was performed on *C. anisus* and *L. segnis* samples using the TIANamp Genomic DNA Kit (TIANGEN, Beijing, China) and following the manufacturer’s instructions.

### PCR amplification

The mitochondrial genomes of *C. anisus* and *L. segnis* were amplified using two sets of overlapping long-fragment RCR primers. Specific PCR primers were constructed from the *Xenopsylla cheopis* (MW310242) and *Pulex irritans* (NC063709) mitochondrial genomes, which correspond to the *COX1* and *12S rRNA* genes, using Primer Premier 5.0 software (CA1: 5′-TTC CCT ACC TGT GCT TGC AG-3′; 3′-AAG AAT TGG ATC TCC CCC GC-5′; CA2: 5′-GCT TGA AAC TTA AAG AAT TTG GCG G-3′, 3′-AAG AGC GAC GGG CAA TAT GT-5′; LS1: 5′-GCA GGA GGG GGT GAT CCT AT-3′; 3′-ATC GTC GAG GTA TTC CTG CT-5′; LS2: 5′-CTT GAA ACT TAA AGA ATT TGG CGG T-3′, 3′-TCC AGT ACA TCT ACT ATG TTA CGA C-5′). The long-range PCR was performed in a total volume of 50 μL, consisting of 10 μL 5× PrimerSTAR GXL Buffer (Takara, Japan), 4 μL of each primer, 4 μL of dNTPs, 1 μL of PrimerSTAR GXL DNA Polymerase (Takara, Japan), 4 μL of DNA template and 23 μL of ddH_2_O under the following conditions: initial denaturation at 92°C for 2 min, followed by 30 cycles of denaturation at 92°C for 10 s, annealing at 68°C for 30 s and extension at 68°C for 10 min and the final extension step was subjected to 68°C for 10 min. The PCR products were tested using 1% agarose gel electrophoresis, which was purified and sequenced by Sangon Biotech Company (Shanghai, China).

### Gene annotation and data analysis

Sequencing on the Illumina NovaSeq platform using AdapterRemoval software to eliminate low-quality data, assembly by software IDBA. The A5-miseq v20150522 program was used to assemble the complete mitochondrial genome and the MITOS WebServer^1^ was used for genome annotation (15). MITOZ tool for mitochondrial genome prediction and online site tRNAscan-SE^2^ for secondary structure prediction of transfer RNA (tRNA) (16, 17). Mitochondrial genome circle mapping with CGView Server.^3^ The software DNAStar V7.1 was used for nucleotide composition analysis and the program CodonW was used to calculate the relative synonymous codon usages (RSCU). The formulas GC-skew = [G – C]/[G + C] and AT-skew = [A – T]/[A + T] were used to measure the relative base content skewness. The total mitogenome informations of *C. anisus* and *L. segnis* have been deposited in NCBI.

### Phylogenetic analysis

Mitochondrial lineages from 15 fleas were determined by phylogeny based on the concatenated datasets of 13 PCGs (Table 1). Phylogenetic trees were constructed using the Bayesian inference (BI) and maximum likelihood (ML) methods in the Mrbayes v.3.2.7 (18) and MEGA 7.0 software, respectively. The BI approach selected GTR + G + I as the best model, running 10,000,000 generations in total, sampling every 1,000 generations. The ML analysis was based on 1,000 bootstrapped, which assessed branch reliability and nodal robustness. The resultant tree was visualized and edited with FigTree v1.4.2 (19).

**Table 1:** List of the 15 flea species and *Casmara patrona* analyzed in this paper with their GenBank numbers.

Species	Family	Length (bp)	Accession number
*Ceratophyllus anisus*	Ceratophyllidae	15,875	OQ366407.1
*Leptopsylla segnis*	Leptopsyllidae	15,785	OQ023576.1
*Xenopsylla cheopis*	Pulicidae	18,902	MW310242.1
*Pulex irritans*	Pulicidae	20,337	NC063709.1
*Ctenocephalides canis*	Pulicidae	15,609	ON109770.1
*Ctenocephalides canis*	Pulicidae	15,609	NC063710.1
*Ctenocephalides felis*	Pulicidae	20,873	NC049858.1
*Ctenocephalides felis*	Pulicidae	20,911	MW420044.1
*Ceratophyllus wui*	Ceratophyllidae	18,081	NC040301.1
*Jellisonia amadoi*	Ceratophyllidae	17,031	NC022710.1
*Jellisonia amadoi*	Ceratophyllidae	17,031	KF322091.1
*Dorcadia ioffi*	Vermipsyllidae	16,785	NC036066.1
*Dorcadia ioffi*	Vermipsyllidae	16,785	MF124314.1
*Hystrichopsylla weida qinlingensis*	Hystrichopsyllidae	17,173	NC042380.1
*Hystrichopsylla weida qinlingensis*	Hystrichopsyllidae	17,173	MH259703.1
*Casmara patrona*	Oecophoridae	15,393	NC053695.1

## Results

### Structure analysis of mitochondrial genome

**Figure 1:**
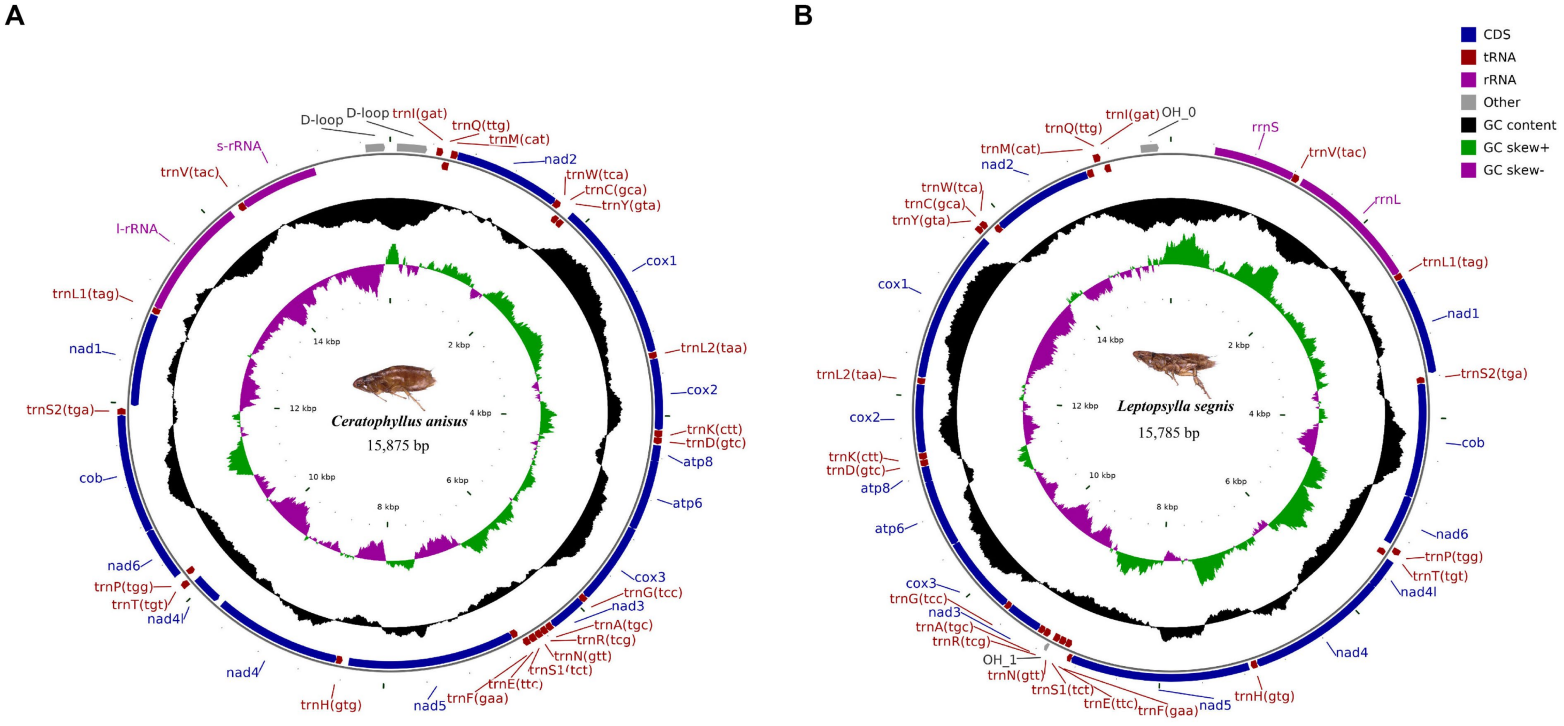
Circular map and organization of the mitochondrial genome of *Ceratophyllus anisus* **(A)** and *Leptopsylla segnis* **(B)**.

The complete mitochondrial genomics of *C. anisus* and *L. segnis* were uploaded to Genbank in TBL format under the accession number OQ366407 and OQ023576. The *C. anisus* and *L. segnis* genomes are circular molecules of 15,875 bp and 15,785 bp in length, respectively, consisting of 13 protein-coding genes, 22 tRNAs, two rRNAs, and two D-loop (Figure 1). Fourteen tRNA genes and nine PCGs are located in the forward strand (+), and the remaining 14 genes are encoded in the reverse strand (−) (Table 2). The average AT content of *C. anisus* and *L. segnis* complete mitochondrial genome is 78.54% (78.89%) and GC content is 21.46% (21.11%), including *A* = 38.41% (40.37%), *T* = 40.14% (38.51%), *G* = 8.25% (13.17%) and *C* = 13.21% (7.94%; Table 3). The mitochondrial genome of *C. anisus* has 18 intergenic regions of 929 bp, accounting for 5.85% of the total length, and 13 overlapping regions totaling 28 bp. The genome of *L. segnis* has 19 spacer areas and 8 overlapping regions with a total of 894 bp and 21 bp (Table 2).

**Table 2:** Organization of the *Ceratophyllus anisus* and *Leptopsylla segnis* mitochondrial genomes.

Gene	Strand	Position	Size(bp)	Initiation codon	Stop codon	Anticodon	Intergenic nucleotide
D-loop	N	1-347/117-287	347/171				91/321
trnI	N	439-501/609-672	63/64			GAT	7/5
trnQ	J	577-509/746-678	69/69			TTG	−1/41
trnM	N	577-643/788-854	67/67			CAT	
nad2	N	644-1657/855-1868	1014/1014	ATT/ATT	TAA/TAA		−2/−1
trnW	N	1656-1722/1868-1932	67/65			TCA	6/7
trnC	J	1789-1729/2001-1940	61/62			GCA	
trnY	J	1853-1790/2062-2002	64/61			GTA	−3/0
cox1	N	1851-3386/2063-3598	1535/1536	ATC/ATC	TAA/TAA		4/4
trnL2	N	3391-3454/3603-3666	64/64			TAA	1/1
cox2	N	3456-4136/3668-4348	681/681	ATG/ATG	TAA/TAA		2/2
trnK	N	4139-4208/4351-4420	70/70			CTT	−1/0
trnD	N	4208-4272/4421-4488	65/68			GTC	9/0
atp8	N	4282-4443/4489-4650	162/162	ATA/ATA	TAA/TAA		−7/−7
atp6	N	4437-5111/4644-5318	675/675	ATG/ATG	TAA/TAA		−1/−1
cox3	N	5111-5893/5318-6100	783/783	ATG/ATA	TAA/TAA		
trnG	N	5894-5956/6101-6159	63/59			TCC	−3/0
nad3	N	5957-6307/6160-6513	350/354	ATA/ATA	TAA/TAA		2/−2
trnA	N	6310-6373/6512-6575	64/64			TGC	−1/−1
trnR	N	6373-6435/6575-6637	63/63			TCG	−3/4
D-loop	N	0/6642-6679	/38				0/3
trnN	N	6433-6497/6683-6747	65/65			GTT	
trnS1	N	6498-6565/6748-6815	68/68			TCT	
trnE	N	6566-6630/6816-6878	65/63			TTC	−3/0
trnF	J	6694-6628/6944-6879	67/66			GAA	−2/2
nad5	J	8410-6693/8660-6947	1717/1714	ATT/ATT	TTA/TTA		61/1
trnH	J	8474-8412/8724-8662	63/63			GTG	−1/0
nad4	J	9810-8474/10063-8725	1337/1339	ATG/ATG	TTA/TTA		59/−7
nad4l	J	10097-9804/10350-10057	294/294	ATG/ATG	TAA/TAA		2/2
trnT	N	10100-10165/10353-10416	66/64			TGT	
trnP	J	10228-10166/10480-10417	63/64			TGG	11/2
nad6	N	10240-10746/10483-10992	507/510	ATT/ATT	TAA/TAA		0/−1
cob	N	10747-11886/10992-12131	1140/1140	ATG/ATG	TAA/TAA		2/2
trnS2	N	11889-11952/12134-12196	64/63			TGA	19/19
nad1	J	12910-11972/13151-12216	938/936	ATT/ATT	TAA/TAA		7/1
trnL1	J	12980-12918/13214-13153	63/62			TAG	0/1
rrnL	J	14198-12981/14489-13216	1218/1274				79/30
trnV	J	14346-14278/14585-14520	69/66			TAC	−1/−1
rrnS	J	15124-14346/15363-14585	779/780				519/420
D-loop	N	15643-15875/0	233/0				48/0

**Table 3:** Composition and skewness of *Ceratophyllus anisus* and *Leptopsylla segnis* mitogenome.

Region	*A*%	*C*%	*G*%	*T*%	*A* + *T*%	*G* + *C*%	AT skew	GC skew
Whole genome	38.41/40.37	13.21/7.94	8.25/13.17	40.14/38.51	78.54/78.89	21.46/21.11	−0.022/0.024	−0.231/0.248
nad2	34.91/35.80	11.14/10.65	7.99/7.00	45.96/46.55	80.87/82.35	19.13/17.65	−0.137/−0.131	−0.165/−0.207
cox1	29.30/29.49	16.47/16.21	13.93/13.15	40.30/41.15	69.60/70.64	30.40/29.36	−0.158/−0.165	−0.084/−0.104
cox2	34.65/33.19	15.42/15.57	10.57/10.72	39.35/40.53	74.01/73.72	25.99/26.28	−0.099/−0.100	−0.187/−0.185
atp8	43.83/40.12	6.17/11.73	4.32/3.70	45.68/44.44	89.51/84.57	10.49/15.43	−0.021/−0.051	−0.176/−0.521
atp6	30.96/33.04	15.26/14.52	9.78/9.04	44.00/43.41	74.96/76.44	25.04/23.56	−0.174/−0.136	−0.219/−0.233
cox3	27.97/29.63	17.11/16.09	13.03/12.77	41.89/41.51	69.86/71.14	30.14/28.86	−0.199/−0.167	−0.135/−0.115
nad3	31.91/31.92	13.11/12.43	7.12/7.34	47.86/48.31	79.77/80.23	20.23/19.77	−0.200/−0.204	−0.199/−0.257
nad5	34.69/34.89	7.16/6.65	13.80/13.54	44.35/44.92	79.05/79.81	20.95/20.19	−0.122/−0.126	0.317/0.341
nad4	33.43/33.76	7.85/7.24	13.84/12.85	44.88/46.15	78.31/79.91	21.69/20.09	−0.146/−0.155	0.276/0.279
nad4l	36.05/34.35	2.72/3.40	13.61/13.61	47.62/48.64	83.67/82.99	16.33/17.01	−0.138/−0.172	0.667/0.600
nad6	35.90/35.10	10.26/11.57	5.72/4.90	48.13/48.43	84.02/83.53	15.98/16.47	−0.146/−0.160	−0.284/−0.405
cob	31.50/31.58	15.35/15.96	11.40/10.53	41.67/41.93	73.25/73.51	26.75/26.49	−0.139/−0.141	−0.148/−0.205
nad1	30.78/33.01	7.14/7.26	15.76/15.49	46.33/44.23	77.10/77.24	22.90/22.76	−0.202/−0.145	0.176/0.362
rrnl	40.48/41.52	6.16/5.73	12.89/12.32	40.48/40.42	80.95/81.95	19.05/18.05	0/0.013	0.353/0.365
rrns	41.98/40.90	6.55/6.41	12.45/12.31	39.02/40.38	81.00/81.28	19.00/18.72	0.037/0.006	0.311/0.315
trnI	41.27/40.62	7.94/7.81	12.70/12.50	38.10/39.06	79.37/79.69	20.63/20.31	0.040/0.020	0.230/0.231
trnQ	40.58/40.58	4.35/4.35	11.59/11.59	43.48/43.48	84.06/84.06	15.94/15.94	−0.034/−0.034	0.454/0.454
trnM	38.81/37.31	17.91/19.40	10.45/13.43	32.84/29.85	71.64/67.16	28.36/32.84	0.083/0.111	−0.263/−0.182
trnW	41.79/43.08	13.43/10.77	7.46/9.23	37.31/36.92	79.10/80.00	20.90/20.00	0.057/0.077	−0.286/−0.077
trnC	44.26/38.71	4.92/8.06	13.11/16.13	37.70/37.10	81.97/75.81	18.03/24.19	0.080/0.021	0.454/0.334
trnY	39.06/42.62	10.94/8.20	18.75/13.11	31.25/36.07	70.31/78.69	29.69/21.31	0.111/0.083	0.263/0.230
trnL2	34.38/32.81	12.50/12.50	14.06/14.06	39.06/40.62	73.44/73.44	26.56/26.56	−0.064/−0.106	0.059/0.059
trnK	32.86/32.86	15.71/15.71	17.14/15.71	34.29/35.71	67.14/68.57	32.86/31.43	−0.021/−0.042	0.044/0
trnD	47.69/50.00	7.69/5.88	9.23/7.35	35.38/36.76	83.08/86.76	16.92/13.24	0.148/0.153	0.091/0.111
trnG	44.44/42.37	6.35/8.47	7.94/11.86	41.17/37.29	85.71/79.66	14.29/20.34	0.038/0.064	0.111/0.167
trnA	37.50/40.32	6.25/9.68	9.38/9.68	46.88/40.32	84.38/80.65	15.62/19.35	−0.111/0	0.200/0
trnR	39.68/34.92	11.11/11.11	11.11/9.52	38.10/44.44	77.78/79.37	22.22/20.63	0.020/−0.120	0/−0.077
trnN	44.62/44.62	7.69/7.69	9.23/9.23	38.46/38.46	83.08/83.08	16.92/16.92	0.364/0.364	0.091/0.091
trnS1	42.65/39.71	10.29/10.29	10.29/10.29	36.76/39.71	79.41/79.41	20.59/20.59	0.281/0	0/0
trnE	41.54/39.68	7.69/9.52	4.62/6.35	46.15/44.44	87.69/84.13	12.31/15.87	−0.053/−0.057	−0.250/−0.120
trnF	35.82/40.91	10.45/6.06	14.93/13.64	38.81/39.39	74.63/80.30	25.37/19.70	−0.040/0.019	0.177/0.385
trnH	42.86/36.51	3.17/3.17	12.70/15.87	41.27/44.44	84.13/80.95	15.87/19.05	0.019/−0.219	0.600/0.667
trnT	42.42/39.06	4.55/6.25	9.09/9.38	43.94/45.31	86.36/84.38	13.64/15.62	−0.018/−0.074	0.333/0.200
trnP	38.10/42.19	4.76/3.12	15.87/14.06	41.27/40.62	79.37/82.81	20.63/17.19	−0.040/0.019	0.539/0.636
trnS2	42.19/42.86	6.25/7.94	12.50/12.70	39.06/36.51	81.25/79.37	18.75/20.63	0.039/0.080	0.333/0.230
trnL1	39.68/38.71	6.35/6.45	12.70/14.52	41.27/40.32	80.95/79.03	19.05/20.97	−0.020/−0.020	0.333/0.385
trnV	43.48/40.91	5.80/7.58	7.25/7.58	43.48/43.94	86.96/84.85	13.04/15.15	0/−0.039	0.111/0
OH	44.31/44.50	2.41/3.35	2.41/11.48	50.86/40.67	95.17/85.17	4.83/14.83	−0.069/0.045	0/0.548

### Protein-coding genes

**Figure 2:**
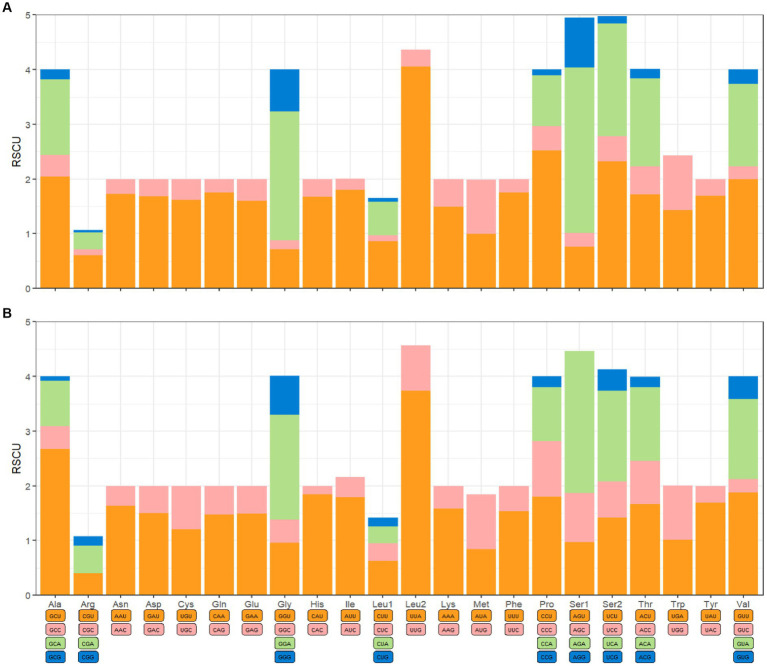
Relative synonymous codon usage (RSCU) of codons. **(A)** *Ceratophyllus anisus* and **(B)** *Leptopsylla segnis*. The horizontal coordinates represent all codons encoding each amino acid, and the vertical coordinates represent the sum of all RECU values.

The mitochondrial genomes of *C. anisus* and *L. segnis* consist of 13 protein-coding genes, with a total length of 11,014 bp and 11,134 bp, accounting for 69.4% and 70.5% of the total length, respectively. The PCGs of *C. anisus* use the standard ATN as the initiation codon, and stop codons are TAA except for *nad5* and *nad4* (TTA). Amino acid utilization and RSCU were calculated for the PCGs of the mitochondrial genomic of *C. anisus* and *L. segnis*, encoding 3,617 and 3,711 amino acids, and the most abundant amino acid was found to be Isoleucine and Phenylalanine, accounting for 9.95% and 9.75%, respectively (Figure 2). The AT-skew and GC-skew of the PCGs of the *C. anisus* range from −0.202 (for *NAD1*) to −0.021 (for *ATP8*) and from −0.296 (for *NAD3*) to 0.320 (for *NAD5*), respectively. Among the 13 protein-coding genes, both *C. anisus* and *L. segnis* had nine genes in the forward chain (*NAD2*, *COX1*, *COX2*, *ATP8*, *ATP6*, *COX3*, *NAD3*, *NAD6*, *Cob*) and four genes in the reverse chain (*NAD1*, *NAD5*, *NAD4*, *NAD4L*), which are consistent with most metazoans such as fleas and ticks (5, 20).

### Transfer RNAs and ribosomal RNAs

The mitochondrial genomes of *C. anisus* and *L. segnis* have 22 tRNA genes and two rRNA genes. The length of 22 tRNAs ranged from 61 bp for *tRNA^Cys^* (59 bp for *tRNA^Gly^*) to 70 bp for *tRNA^Lys^* (70 bp for *tRNA^Cys^*), with a total length of 1,433 bp (1,397 bp). The tRNA genes of both samples can form a complete typical canonical cloverleaf structure. There is an overlap between *ATP8* and *ATP6* with a length of 7 bp, which is typical of arthropods (21). The *16S rRN*A and *12S rRNA* genes of the *C. anisus* and *L. segnis* were separated by *Valine*, and were 1,218 bp (1,274 bp) and 779 bp (780 bp) in length, respectively (Table 2), a structure consistent with that reported in the mitogenome of other flea species (22).

### Phylogenetic analysis

**Figure 3:**
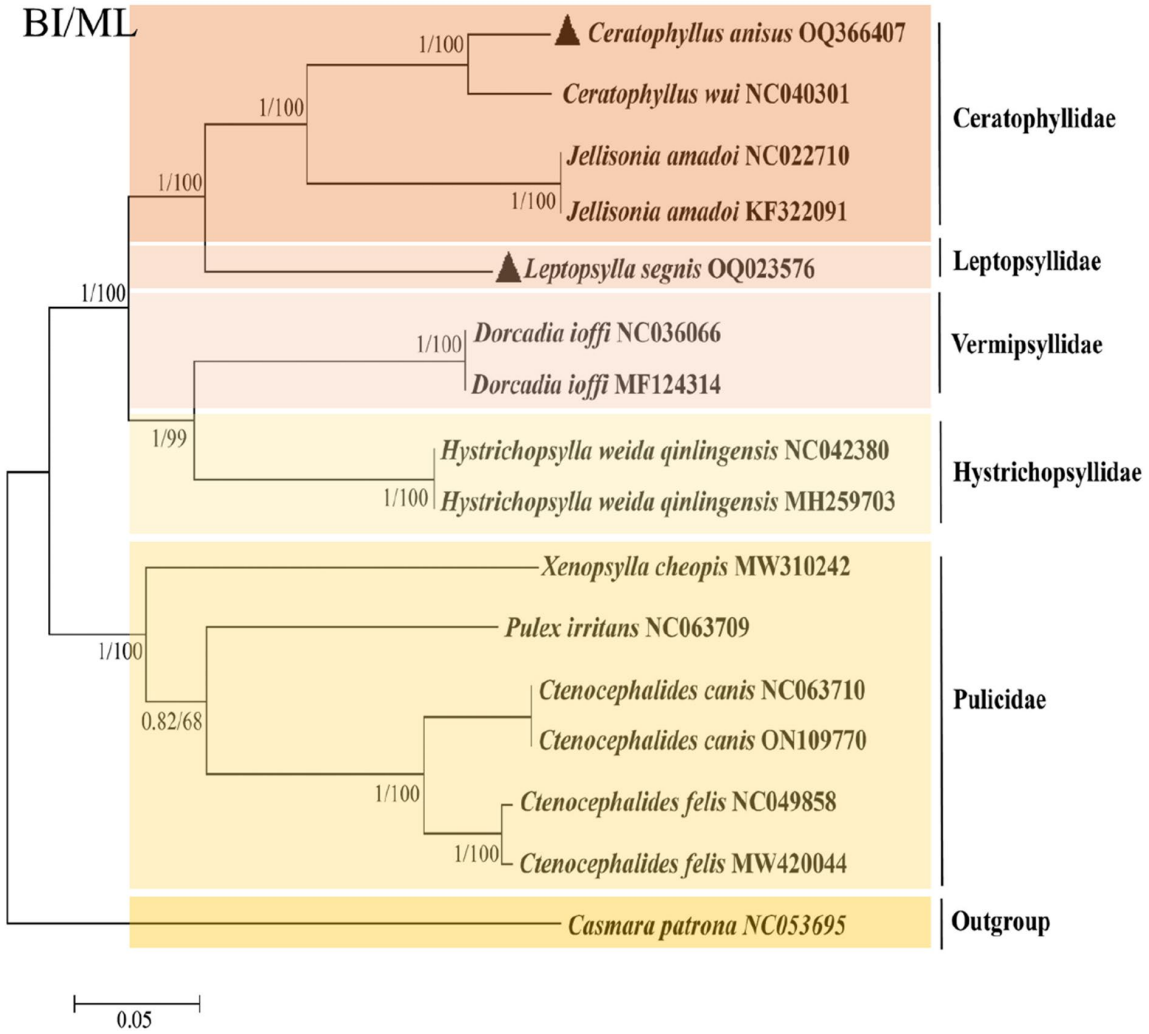
Phylogenetic analysis based on the nucleotide sequences of the 13 PCGs in the mitogenome. Each genus is represented by different colors. The solid black triangle represents the species in this study.

To further analyze the phylogenetic relationships of fleas, we added the mitochondrial genomes of *C. anisus* and *L. segnis* to the analysis. Phylogenetic trees were constructed using the BI and ML methods for the concatenated nucleotide sequences of 13 PCGs of the mitochondrial genome of 15 fleas and *Casmara patrona* as an outgroup, and the topologies of the two methods were consistent. According to the topology analysis, *C. anisus* and *Ceratophyllus wui* are clustered in a branch with high statistical support and *L. segnis* is alone in a branch, forming a sister group with other families of fleas (Figure 3). The families Ceratophyllidae, Leptopsyllidae, Vermipsyllidae, Hystrichopsyllidae, and Pulicidae form monophyletic branches, which is consistent with the previous findings (23).

## Discussion

As a temporary host and vector of some important human infectious diseases such as plague and endemic typhus, fleas are early warning indicators for judging the prevalence of plague and other human-animal infectious diseases (24). In recent years, plague has rebounded in some regions, with an increasing trend in incidence, which has always been a persistent and difficult problem worldwide. *C. anisus* and *L. segnis* are common species of fleas that play an important role in the transmission of zoonotic diseases.

The D-loop region has low evolutionary pressure, a large number of gene rearrangements, and rapid base substitutions, making it an effective molecular marker for population genetic studies. The frequency and location of the D-loop vary from species to species and tissue to tissue, and its length is influenced by the number of tandem repeat copies, which in turn affects the length of the entire mitogenome (25). One D-loop region was found for *X. cheopis*, *P. irritans*, and *C. wui*, and two D-loops existed for *Ctenocephalides felis*, *C. anisus*, and *L. segnis*. The two control regions were also found in some ticks and sea cucumbers, and the mtDNA was replicated more efficiently, so it is speculated that the two D-loop regions acted synergistically during the evolutionary process (26). The mtDNAs of *C. anisus* and *L. segnis* have the same gene composition and arrangement as that of most flea species. Bases mismatch appears in most tRNA genes, and G-U wobble base pairs conform to the oscillating pairing principle, which is very important for maintaining the stability of the tRNA secondary structure (27).

The phylogenetic tree derived using all flea mitochondrial genomic data in the NCBI gene bank shows that the five families are divided into two distinct branches, with Ceratphyllidae, Leptosyllidae, Vermipsyllidae, and Hystrichopsyllidae clustered into one, and the Pulicidae family as the other branch. The branch where *L. segnis* is located and the *C. anisus* and *C. wui* branches form a sister group with high node support. The same species of fleas from different hosts and different geographical locations are clustered together in this phylogenetic tree with posterior probabilities and bootstrap values of 1 and 100, respectively, with a high degree of confidence.

The mtDNA is a valuable marker for population biology, species identification classification, and phylogenetic studies, especially for assessing genetic diversity and identifying cryptic species as well as population structure. There are still gaps in molecular data for *C. anisus* and *L. segnis*, which is a major obstacle to the development of flea species. In this study, we obtained the complete mitochondrial genome, which provides more accurate evidence for the phylogenetic relationships of flea species. To better understand the phylogenetic relationship among fleas, the mitochondrial genome study within the Siphonaptera order must be expanded. We expect that the complete mitogenomes of *C. anisus* and *L. segnis* will provide important genome information for molecular phylogenetic studies and contribute to clarifying the phylogeny and evolution of Siphonaptera.

## Conclusion

In this study, the complete mitochondrial genomes of *C. anisus* and *L. segnis* are sequenced and annotated for the first time by long-range PCR combined with Illumina sequencing technology which will be helpful for future research on fleas. The results of this study contributed to the fleas, filling the flea mitochondrial genome database resources and laying the foundation for further understanding the phylogenetic relationships of the fleas. With the development of molecular biology, sequencing techniques using mitochondrial genomes as molecular markers have effectively bridged the morphological gap and have been widely used in species identification, kinship, and evolutionary studies.

## Data availability statement

The datasets presented in this study can be found in online repositories. The names of the repository/repositories and accession number(s) can be found in the article/supplementary material.

## Ethics statement

The animal study was reviewed and approved by Laboratory Animal Management Committee of Dali University and First Affiliated Hospital of Chengdu Medical College.

## Author contributions

YL conceived the study and wrote the manuscript. BC, XL, and DJ carried out the experiments and analysed the data. TW and LG contributed to the collection of *C. anisus* and *L. segnis* and discussions. QZ and XY is responsible for the interpretation of experimental data, critical revision of important knowledge content, and final approval of the version to be published. All authors contributed to the article and approved the submitted version.

## Funding

This work was supported by the Yunnan Natural Science Foundation (2017FD139) and Scientific Research Fund of Yunnan Education Department (2022J0687).

## Conflict of interest

The authors declare that the research was conducted in the absence of any commercial or financial relationships that could be construed as a potential conflict of interest.

## Publisher’s note

All claims expressed in this article are solely those of the authors and do not necessarily represent those of their affiliated organizations, or those of the publisher, the editors and the reviewers. Any product that may be evaluated in this article, or claim that may be made by its manufacturer, is not guaranteed or endorsed by the publisher.

## Footnotes

^1^ http://mitos.bioinf.uni-leipzig.de/index.py

^2^ http://lowelab.ucsc.edu/tRNAscan-SE/

^3^ https://paulstothard.github.io/cgview/
